# Population Connectivity and Phylogeography of a Coastal Fish, *Atractoscion aequidens* (Sciaenidae), across the Benguela Current Region: Evidence of an Ancient Vicariant Event

**DOI:** 10.1371/journal.pone.0087907

**Published:** 2014-02-20

**Authors:** Romina Henriques, Warren M. Potts, Carmen V. Santos, Warwick H. H. Sauer, Paul W. Shaw

**Affiliations:** 1 Centre for Ecology, Evolution and Behavior, School of Biological Sciences, Royal Holloway University of London, Egham, United Kingdom; 2 Department of Ichthyology and Fisheries Science, Rhodes University, Grahamstown, South Africa; 3 Faculdade de Ciências, Universidade Agostinho Neto, Luanda, Angola; 4 Institute of Biological, Environmental and Rural Sciences (IBERS), Aberystwyth University, Aberystwyth, United Kingdom; George Washington University, United States of America

## Abstract

Contemporary patterns of genetic diversity and population connectivity within species can be influenced by both historical and contemporary barriers to gene flow. In the marine environment, present day oceanographic features such as currents, fronts and upwelling systems can influence dispersal of eggs/larvae and/juveniles/adults, shaping population substructuring. The Benguela Current system in the southeastern Atlantic is one of the oldest upwelling systems in the world, and provides a unique opportunity to investigate the relative influence of contemporary and historical mechanisms shaping the evolutionary history of warm-temperate fish species. Using the genetic variation in the mitochondrial DNA Control Region and eight nuclear microsatellite DNA loci, we identified the presence of two highly divergent populations in a vagile and warm-temperate fish species, *Atractoscion aequidens*, across the Benguela region. The geographical distributions of the two populations, on either side of the perennial upwelling cell, suggest a strong correlation between the oceanographic features of the system and the breakdown of gene flow within this species. Genetic divergence (mtDNA *φ*_ST_ = 0.902, microsatellite *F*_ST_ = 0.055: probability of genetic homogeneity for either marker = *p*<0.001), absence of migrants (less than 1% per generation) between populations and coalescent estimates of time since most recent common ancestor suggest that the establishment of the main oceanographic features of the system (2 million years ago), particularly the strengthening and position of the perennial upwelling cell, is the most likely mechanism behind the observed isolation. Concordance between mitochondrial and nuclear genetic markers indicates that isolation and divergence of the northern and southern Benguela populations of *A. aequidens* occurred deep in the past and has continued to the present day. These findings suggest that the Benguela Current system may constitute an ancient and impermeable barrier to gene flow for warm-temperate fish species.

## Introduction

Contemporary patterns of genetic diversity and population connectivity within species are known to be influenced both by historical population processes, and by historical and present barriers to gene flow [1]. In the marine environment, present day barriers to adult and/or larval dispersal (and so gene flow) may constitute such features as different spawning grounds [2], geographical distances [3], oceanographic frontal systems [4], [5], [6], upwelling cells [7], [8] and environmental transitions [9]. Similarly, historical climatic changes during the Pleistocene which resulted in fluctuation in sea surface temperatures, sea level, ice sheet coverage and oceanographic circulation patterns have been linked to significant changes in demographic history [10], distribution patterns [11], genetic diversity [12], population substructuring [13] and speciation events [14], [15].

The Benguela Current, in the southeastern Atlantic, is characterized by cold sea surface temperatures, high productivity levels, and the presence of a perennial upwelling cell that physically divides the system into two contrasting subsystems [16], [17], [18]. This oceanographic system is bounded to the north and south by the warm, fast-flowing Angola and Agulhas currents, respectively, creating warm-temperate confluence zones [16], [17], [18]. Although the Benguela Current first formed in the mid-Miocene (12–10 Million years ago – Ma) [19], [20], its present day features were established by changes in Atlantic Oceanic circulation patterns resulting from uplift of the Isthmus of Panama and the increase in ice sheet coverage in both northern and southern hemispheres during the Pliocene-Pleistocene transition (2 Ma) [21], [22]. Three major events are known to have contributed to environmental changes in the region: i) intensification of the upwelling regime during the Pliocene-Pleistocene transition (2 Ma); ii) severe cooling due to the increase of ice sheet coverage in the mid-Pleistocene (1–0.5 Ma); and iii) establishment of the contemporary Quaternary 40–100 thousand year (Ky) glacial-interglacial cycles (0.5–0.01 Ma) [22], [23], [24]. Such climatic fluctuations are likely to have contributed to significant changes in demographic histories and population connectivity of coastal fish species. Recent studies have demonstrated that several coastal fish species from the southern Benguela subsystem have experienced population growth dating from the end of the last glacial maximum (LGM) circa 20 thousand years ago (Ka) [25], [26], [27], [28]. The historical climatic changes in the Benguela Current, coupled with distinct large scale oceanographic features make this system ideal to compare the influence of contemporary and historical factors on the evolutionary history of marine fishes. However, no comprehensive studies have been conducted for species with distributions in both the northern and southern warm-temperate zones.

*Atractoscion aequidens* (Cuvier 1830) is a migratory, benthopelagic sciaenid fish inhabiting coastal environments (15–200 m depth) along the western coast of Africa, from Mauritania to South Africa, and the eastern coast of Australia [29], [30]. In the southeastern Atlantic, the distribution of this species coincides with the warm-temperate environments created by the confluence of the cold Benguela Current with the tropical Angola and Agulhas Currents. Migration between these two regions is considered to be negligible [29]. This disjunct distribution, along with a high dispersal potential during the egg/larval and adult phases, make *A. aequidens* an ideal candidate to investigate the influence of oceanographic features on population connectivity. Because demographic changes cannot be inferred from climatic or fossil records, genetic data can provide a valuable source of information in this context. To uncouple the influence of contemporary versus historical processes, we analyzed both mitochondrial DNA (mtDNA) and nuclear microsatellite DNA variation in *A. aequidens* to investigate: i) present patterns of genetic diversity; and ii) the influence of Pleistocene climatic events on genetic connectivity, phylogeography and demographic history across the Benguela Current system. The data presented here provides the first comprehensive study of the influence of the Benguela Current oceanographic features in the genetic substructuring of coastal marine fish species in the region.

## Methods

### Ethics Statement

Due to the nature of the sampling effort, fin clipping from commercial fishing catches, no ethics approval was considered necessary. Permissions for collecting samples were obtained when necessary (Faculdade de Ciências, Universidade Agostinho Neto, Angola; and Rhodes University, South Africa).

### Sampling

**Figure 1:**
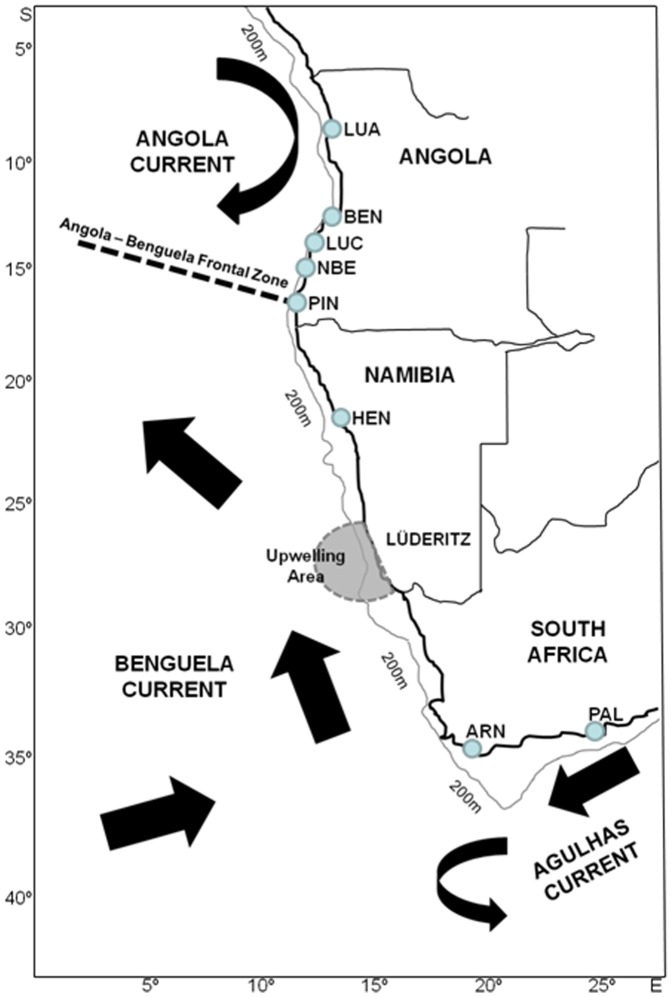
Oceanographic regions sampled. Sampling strategy for *A. aequidens* across the Benguela Current region, highlighting sampling sites (see Table 1 for sampling codes), and their position relative to the major oceanographic features of the system: position of the Benguela and Agulhas Currents, central Namibia upwelling cell, and continental platform width.

A total of 558 fish were collected throughout the Benguela Current region during 2008–2010: Angola (*n* = 382 – sampling sites LUA, BEN, LUC, NBE, PIN), northern Namibia (*n* = 7 – HEN) and South Africa (*n* = 169 – PAL, ARN) (see Table 1 and Figure 1). All individuals were collected from local fishermen, and a fin clip removed and stored in 95% ethanol. Total genomic DNA was extracted from fin clips using a standard phenol/chlorophorm method [31].

**Table 1:** Sampling strategy for *A. aequidens* across the Benguela Current region: sampling locations, sample code and sample size.

Country	Benguela subsytem	Site	Code	Sample size
		Luanda	LUA	59
		Baía Farta	BEN	24
**Angola**	Northern	Lucira	LUC	109
		Namibe	NBE	79
		Pinda	PIN	99
**Namibia**	Northern	Henties Bay	HEN	9
**South Africa**	Southern	Arniston	ARN	90
		Port Alfred	PAL	79

### Genotyping

Genetic variation was screened at one mitochondrial DNA (mtDNA) region and eight nuclear microsatellite loci (Table 2). The mtDNA Control Region (CR) was amplified by polymerase chain reaction (PCR) for 7–20 fish per sampling site (Table 2) using universal primers and protocols from [32]. Obtained PCR products were purified following the protocol of [8], and sequenced by Macrogen, Inc. (South Korea) with the same primers. DNA sequences were visually inspected and aligned with CLUSTAL X [33] in BioEdit v.7.0.1.

**Table 2:** Mitochondrial genetic diversity within the Control Region (CR) of *A. aequidens* from sites (see Table 1 for codes) within the northern and southern Benguela subsystems: n – number of individuals; H – number of haplotypes; PH – number of haplotypes private to sites within subsystems; *h* – haplotype diversity; *π* - nucleotide diversity.

	LUA	BEN	LUC	NBE	PIN	HEN	Overall Northern	ARN	PAL	Overall Southern
**n**	20	17	20	20	20	7	**104**	20	20	**40**
**H**	9	9	9	8	11	4	**32**	10	13	**19**
**PH**	4	5	3	4	4	2	**23**	6	9	**15**
***h***	0.826	0.860	0.837	0.805	0.921	0.8095	**0.853**	0.837	0.932	**0.901**
*π*	0.010	0.010	0.009	0.006	0.008	0.0046	**0.005**	0.008	0.010	**0.008**

A total of 395 individuals from six different localities (Table 3) were screened for genetic variation at microsatellite markers, designed specifically for *A. aequidens* [34]. PCR products were genotyped on an AB3500 Genetic Analyzer (Applied Biosystems), with alleles scored against an internal size marker (LIZ-600) as PCR product size in base pairs, using GeneMapper v.4.0 (ABIPrism). In order to ensure accurate allele size scoring between runs, individuals with known allele sizes were used in each run as positive controls. Allelic and genotypic frequencies within samples were tested for deviations from Hardy-Weinberg outcrossing expectation within loci and for linkage equilibrium between loci using Genepop v.3.4 [35]. Sequential Bonferroni corrections were used for multiple tests with *p*<0.05 [36]. Amplification errors, such as large allele drop out and stuttering were assessed in MICROCHECKER v.2.2.3 [37], while null allele frequencies were estimated in FreeNA [38].

**Table 3:** Genetic diversity at eight microsatellite loci in *A. aequidens*: n – number of individuals genotyped; Na – number of alleles; AR – allelic richness for a minimum of 47 individuals; H_E_ – expected heterozygosity; H_O_ – observed heterozygosity; F_IS_ – inbreeding coefficient (significant deviations to Hardy-Weinberg expectations in bold, *p*<0.05).

		LUA	LUC	NBE	PIN	ARN	PAL
	**n**	48	70	66	70	70	69
	**Na**	22	23	26	25	24	25
	**AR**	21.916	21.684	23.967	22.749	21.839	23.206
**Geelb5**	**H_E_**	0.928	0.918	0.932	0.928	0.898	0.930
	**H_O_**	0.958	0.971	0.894	0.929	0.900	0.855
	**F_IS_**	−0.022	−0.051	0.039	0.007	0.005	**0.088**
	**n**	47	70	68	70	70	70
	**Na**	7	10	9	8	4	6
	**AR**	7.000	9.001	8.260	7.626	4.00	5.562
**Geelb13**	**H_E_**	0.678	0.669	0.645	0.680	0.641	0.644
	**H_O_**	0.681	0.617	0.691	0.729	0.643	0.614
	**F_IS_**	0.002	0.003	−0.065	−0.065	0.004	0.053
	**n**	48	70	67	70	70	70
	**Na**	20	20	21	20	21	23
	**AR**	19.937	18.846	19.800	18.722	19.401	20.958
**Geelb16**	**H_E_**	0.927	0.926	0.925	0.929	0.927	0.929
	**H_O_**	0.875	0.957	0.955	0.971	0.800	0.943
	**F_IS_**	0.067	−0.026	−0.025	−0.039	**0.144**	−0.088
	**n**	47	70	68	70	70	70
	**Na**	20	24	21	22	19	21
	**AR**	20.000	21.960	20.010	19.746	16.887	18.436
**Geelb21**	**H_E_**	0.894	0.881	0.882	0.881	0.902	0.910
	**H_O_**	0.808	0.843	0.897	0.871	0.927	0.943
	**F_IS_**	0.106	0.051	−0.009	0.018	−0.022	−0.029
	**n**	47	70	68	70	70	70
	**Na**	20	21	19	22	19	19
	**AR**	20.000	19.107	18.750	20.059	17.404	17.747
**Geelb27**	**H_E_**	0.927	0.927	0.933	0.928	0.916	0.918
	**H_O_**	0.957	0.900	0.912	0.929	0.971	0.957
	**F_IS_**	−0.022	0.036	0.030	0.006	−0.053	−0.036
	**n**	48	70	68	70	70	70
	**Na**	17	18	20	22	39	32
	**AR**	16.937	16.867	19.006	19.590	34.618	29.066
**Geelb29**	**H_E_**	0.916	0.918	0.922	0.921	0.960	0.946
	**H_O_**	0.899	0.857	0.882	0.914	0.927	0.627
	**F_IS_**	0.033	0.074	0.050	0.014	0.040	**0.342**
	**n**	48	69	68	70	70	70
	**Na**	19	17	20	22	19	21
	**AR**	18.916	15.921	18.508	19.630	18.038	19.134
**Geelb30**	**H_E_**	0.917	0.915	0.920	0.924	0.925	0.923
	**H_O_**	0.896	0.899	0.926	0.886	0.900	0.900
	**F_IS_**	0.033	0.025	0.000	0.048	0.034	0.032
	**n**	48	70	66	70	69	70
	**Na**	40	49	43	49	51	49
	**AR**	39.685	42.176	38.198	42.868	43.989	40.699
**Geelb32**	**H_E_**	0.965	0.968	0.965	0.9710	0.971	0.965
	**H_O_**	0.896	0.857	0.879	0.871	0.971	0.871
	**F_IS_**	0.082	**0.121**	0.097	0.110	0.008	0.104
	**n**	70	70	66	70	70	70
	**Na**	21	23	22	24	24	24
	**AR**	20.549	20.695	20.812	21.374	22.022	21.881
**Average all loci**	**H_E_**	0.894	0.890	0.889	0.895	0.893	0.896
	**H_O_**	0.871	0.869	0.880	0.887	0.880	0.839
	**F_IS_**	0.036	**0.031**	**0.018**	0.016	0.021	**0.070**

### Population substructuring of *A. aequidens* in the Benguela Current region

Number of haplotypes (*H*), private haplotypes (*PH*), haplotype diversity (*h*) and nucleotide diversity (*π*) were calculated for mtDNA CR sequences for each sample location using Arlequin v.3.5.1 [39]. Evaluation of genetic variability within and among sample locations was conducted using different approaches. First, pairwise *φ*_ST_ values between samples were estimated using Arlequin v.3.5.1 [39], with significance at the 0.05 level determined by 10,000 permutations. Second, a global analysis of hierarchical molecular variance (AMOVA) was performed, as implemented in Arlequin v.3.5.1 [39], with distances calculated using the Kimura 80 (K80) model as selected by jModeltest v.0.1.1 [40], following the Akaike Information Criterion, to specifically test for differentiation between northern and southern Benguela subsystem populations of *A. aequidens*.

From the microsatellite dataset, intraspecific and within-population nuclear genetic diversity was estimated as number of alleles (*N_a_*), allelic richness (*AR*), observed and expected heterozygosity (*H_O_* and *H_E_*), and Wright's inbreeding coefficient (*F_IS_*), as implemented in FSTAT v.2.9.3 [41]. Evaluation of the statistical power of the microsatellite dataset to detect genetic divergence was conducted in Powsim [42]. Population genetic drift was simulated to *F*_ST_ levels of 0.005, 0.02 and 0.05 using two effective population sizes (*N_e_* = 500 and *N_e_* = 2000), and varying the number of generations (*t*) accordingly. As the software only supports up to 50 alleles per locus, locus Geelb32 was removed (88 alleles), and all simulations performed for seven loci and six populations (*n* = 50 to *n* = 70). Each simulation was run for 1,000 replicates, and power was estimated as the proportion of the 1,000 tests that indicated significant genetic differentiation. After assessing power of the microsatellite dataset, pairwise population genetic differentiation was estimated using Weir & Cockerham's *F_ST_* estimator [43], as implemented in FSTAT v.2.9.3 [41]. Marine fish populations often exhibit high genetic diversity so values of *F_ST_* can be low [44]. Therefore, genetic differentiation between samples was also estimated using Jost's *D*_est_, which is independent of heterozygosity, in SMOGD v.1.2.5 [45]. Hierarchical analyses of molecular variance were performed in Arlequin v.3.5.1 [39], to evaluate the population substructuring hypothesis tested for the mtDNA dataset. In addition, a Bayesian approach was used to investigate population substructuring without prior knowledge of geographical origin of individuals, as implemented in STRUCTURE v.2.2.3 [46]. Simulations were conducted under the admixture model, with correlated allele frequencies, for a given number of inferred clusters, *K*, from *K* = 1–6, using burn-in lengths of 20,000 and run lengths of 80,000 Monte Carlo Markov Chain (MCMC) steps. In each analysis, five independent runs were performed for each value of *K* to ensure convergence of parameters during the runs. Estimation of the most likely *K* was performed based on the obtained posterior probabilities of the data [47].

### Phylogeography and demographic history of *A. aequidens* in the Benguela Current region

Haplotype networks were constructed to evaluate intraspecific relationships among all haplotypes identified, using the Median-Joining (MJ) algorithm implemented in Network v.4.6 [48]. The shortest tree was chosen using the coalescent theory approach, where haplotypes should be preferentially linked to the most abundant and/or geographically closest occurring haplotype [11].

Assessment of past population expansion was conducted on the mtDNA dataset using Tajima's *D* [49] and Fu's *FS* [50] for each identified genetic cluster, in Arlequin v. 3.5.1 [39]. If significant population growth was detected, population size before expansion (*θ_0_* = 2N_0_μ), size after expansion (*θ_1_* = 2N_1_μ) and number of generations elapsed since expansion (*τ* = 2μt, where *μ* is the substitution rate per My, and *t* time in My) were estimated assuming a sudden expansion model, and applying a general CR sequence divergence rate of 3.6% per My for marine teleosts [51]. Generation time was estimated at 2.2 years for Angolan/Namibian fish (W. Potts, unpubl.) and 5 years for South African fish [30]. The generation time, in this case defined as the average age at which females reproduce for the first time, was over twice as South African population (5 years, than for the Angolan-Namibian population (2.2 years, W. Potts, unpubl.). Although the reason for the different generation times remains uncertain, it has been suggested that large, migratory fishes often have delayed maturity, since the development of large body sizes, commonly associated with migratory movements [52], requires that energy is invested away from reproduction. Based on the temporal and spatial patterns in catch composition, it is suggested that the South African population of *A. aequidens* undertakes large-scale migrations [30]. While an extensive catch-based survey was not available throughout its distribution for the Angola-Namibian population, no similar trend was observed at a major spawning ground in southern Angola (W. Potts, unpubl.), suggesting that this population does not undertake a large-scale migration, and may explain its smaller maximum size, size at maturity and, subsequently, generation time. In addition, Bayesian skyline plots, implemented in BEAST v.1.7.4 [53], were performed to explore demographic changes through time within each putative population. Skyline plots employ coalescent theory to reconstruct fluctuation through time in effective population size (*N_e_*) using DNA sequence variation, when the nucleotide substitution model and rate are known [54]. The appropriate model was estimated for each population in jModeltest [40]. Two independent runs were performed, using the piece-wise constant method for population expansion, for 50 million MCMC generations, sampling every 5,000 generations. Convergence and visualization of median and 95% highest posterior probability density intervals (HPD) were assessed in Tracer v.1.5 [55], using the Effective Sample Size (ESS>200) as an indicator. As with the *D* and *FS* tests, in order to date the fluctuation of female effective population size (*N_ef_*) through time, a strict molecular clock was enforced, using a CR sequence divergence rate of 3.6% per My.

Patterns of average, historical and contemporary connectivity between the northern and southern subsystem populations (migration rate, *m*), as well as *N_e_* and *N_ef_* were estimated using the coalescent-based method in Migrate v.3.4.2 [56]. For both mtDNA and microsatellite datasets three replicates were run using a Bayesian approach, and enforcing the full migration model [56]. Each analysis was performed with four connected chains, using static heating (temperatures for mtDNA and microsatellites were: 1, 1.5, 3, 6 and 1, 2.38, 4.89, 93,807.14, respectively), and a burn-in period of 25,000 steps, followed by 100,000 steps with parameters recorded every 100 steps. Estimates of *m* were obtained from *M* (*M* = mμ), and estimates of *N_e_* and *N_ef_* were obtained from *θ* (*θ* = xN_e_μ, where x = 4 for microsatellites, and x = 1 for mtDNA). Inference of immigration rate per generation was obtained by multiplying *M* and *θ* (xN_e_m) [56]. In order to obtain estimates of migration rates per generation (and not scaled by mutation) a general divergence rate was applied for mtDNA CR (3.6% per My) [51], but as there is no consensus regarding the mutation rate for microsatellites, estimates of *m* were obtained by applying a range of mutation rates: the commonly used 0.1% per generation, but also 0.05% and 0.2% per generation. In addition, in order to estimate recent migration rates (1–2 generations) between the two populations, the coalescent approach implemented in BAYESASS [57] was used. Three independent runs were performed for 1,000,000 iterations, with a burn-in period of 10,000, and parameters recorded every 100^th^ iteration. Convergence of runs was assessed in Tracer v.1.5 [55].

In order to untangle the influence of contemporary and historical factors in shaping population substructuring, a coalescent approach was employed to estimate time since most recent common ancestor (tmrca) from the mtDNA dataset. Two independent runs (10 million MCMC generations, sampling every 1,000 generation), using the HKY nucleotide substitution model and establishing the tree prior as the coalescent with constant size, were conducted in BEAST v.1.7.4 [53]. Convergence of run parameters (ESS>200), tmrca and 95% HPD intervals were recorded in Tracer v.1.5 [55]. Calibration of the molecular clock was conducted by fixing the lineage divergence rate at 1.8% (3.6% divergence rate per My) [51] and by using major climatic events in the Benguela Current region as biogeographical calibrators: i) the Pliocene-Pleistocene transition, with the establishment of present day features of the system (2 Ma); ii) the mid-Pleistocene abrupt cooling (1 Ma); and iii) the more recent glacial-interglacial cycles during the Quaternary (0.5–0.01 Ma) [22], [23], [24]. Biogeographical calibration was conducted by applying an exponential prior [58], with the mean and zero offset calculated to centre on the appropriate dates: i) 2.5 Ma and 1.5 Ma; ii) 0.5 Ma and 0.5 Ma; iii) 0.1 Ma and 0.02 Ma, respectively. Assessment of the most likely event was performed based both on the comparison of likelihood values for each model, using the Bayes Factor (BF) analyses, and on the estimated divergence rate in each simulation, in Tracer v.1.5 [55].

## Results

### Genetic variation

For the mtDNA dataset 97 Angolan, 7 Namibian and 40 South African *A. aequidens* individuals (GenBank accession number JX192142-286) were amplified and sequenced for 583 bp of CR. The 144 sequences yielded 51 haplotypes, defined by 60 variable nucleotide sites, 51 of which sites were parsimony informative and 29 representing fixed differences between samples from Angola/Namibia and South Africa (Table 2). Of the 51 haplotypes, 32 were restricted to the Angolan and Namibian samples (northern haplogroup), and the remaining 19 found in South African samples (southern haplogroup) only (Table 2). Overall, Angolan *A. aequidens* exhibited high levels of haplotype diversity (*h* = 0.853) and moderate levels of nucleotide diversity (*π* = 0.005), with consistent levels across samples (*h* = 0.805 to 0.921, and *π* = 0.006 to 0.010). Despite a smaller sample size, the southern haplogroup exhibited slightly higher genetic diversity values (*h* = 0.901, *π* = 0.008) (Table 2).

Microsatellite genotypes were obtained for 235 Angolan and 160 South African *A. aequidens* individuals (doi:10.5061/dryad.5gf80), and did not exhibit evidence of amplification errors, conforming to Hardy-Weinberg and linkage equilibrium expectation for the majority of loci and samples (Table 3). Samples from LUC, NBE and PAL exhibited significant positive *F*_IS_ due to heterozygote deficits at loci Geelb5, Geelb29 and Geelb32 respectively, ascribed to frequencies of null alleles (<5% in Geebl5 and Geelb32, 16% for Geelb29) below the level accepted to have no substantial impact on differentiation statistics [59]. Samples from NBE and PIN exhibited significant departures from linkage equilibrium between loci Geelb13/Geelb21/Gellb30 and Geelb13/Geelb27/Geelb30 respectively. As there was no consistent pattern across samples or loci, and no evidence of linkage disequilibrium when all Angolan or South African samples were pooled together, these few deviations were considered to be chance statistical effects with no significant implication for global genetic diversity or differentiation patterns. Levels of nuclear genetic diversity within single loci and across multiple loci, as assessed by number of alleles (*N_a_*), allelic richness (*AR*) and expected heterozygosity (*H_E_*), were similarly high across all samples (Table 3). Assessment of the power of the multilocus dataset to detect differentiation indicated that seven of the eight loci used could accurately detect differentiation as low as *F*_ST_ = 0.005, for a population sample of *n* = 50, indicating that the dataset was suitable for population differentiation inference.

### Population substructuring of *A. aequidens*

There was significant genetic differentiation between *A. aequidens* samples, both for the mtDNA (*φ*_ST_ = 0.902, *p*<0.001) and microsatellite (*F*_ST_ = 0.055, p<0.001) datasets. For mtDNA, pairwise tests (Table 3) indicated significantly high *φ*_ST_ in all comparisons between samples from Angola/Namibia and South Africa (average *φ*_ST_ = 0.890, *p*<0.001). No significant differentiation was observed among Angolan and Namibian samples (overall *φ*_ST_ = 0.035, *p*>0.05) or among South African samples (*φ*_ST_ = 0.019, *p*>0.05).

A similar pattern of genetic substructuring was observed in the microsatellite dataset, with all samples from Angola being significantly different from all samples from South Africa (global *F*_ST_ = 0.055, *p*<0.05; pairwise *F*_ST_ = 0.006–0.05, all *p*<0.001), but no significant differentiation within each area (Angola overall *F*_ST_ = 0.005, *p*>0.05; South Africa overall *F*_ST_ = 0.003, *p*>0.05) – see Table 4. Estimates of population differentiation obtained with Jost's *D*_est_ (Table 4) were double those obtained with *F*_ST_ (overall *D*_est_ = 0.035, *p*<0.05), and ranged from *D*_est_ = 0 (among Angolan samples, *p*>0.05) to *D*_est_ = 0.268 (between NBE and PAL, *p*<0.001).

**Table 4:** Genetic differentiation (*φ*_ST_) between *A. aequidens* samples based on mtDNA CR sequences (below diagonal) and eight nuclear microsatellite loci (above diagonal: *F*_ST_ (*D*_est_)).

	LUA	BEN	LUC	NBE	PIN	HEN	ARN	PAL
**LUA**	-	-	0.000(0.000)	0.001(0.005)	0.009(0.011)	-	**0.056(0.188)**	**0.050(0.181)**
**BEN**	−0.031	-	-	-	-	-	-	-
**LUC**	0.004	−0.014	-	0.000(0.000)	0.008(0.009)	-	**0.058(0.206)**	**0.052(0.194)**
**NBE**	0.023	0.003	−0.005	-	0.008(0.004)	-	**0.060(0.241)**	**0.056(0.268)**
**PIN**	0.095	0.071	−0.001	0.059	-	-	**0.052(0.214)**	**0.058(0.191)**
**HEN**	−0.009	−0.036	0.012	0.025	0.088	-	-	-
**WestC**	**0.879**	**0.882**	**0.885**	**0.903**	**0.888**	**0.876**	-	0.003(0.011)
**EastC**	**0.888**	**0.891**	**0.894**	**0.911**	**0.896**	**0.889**	0.031	-

Values significantly greater than zero (*p*<0.05) in bold.

Hierarchical analyses of the distribution of genetic variance (AMOVA) corroborated the pairwise sample tests, in finding significant variation distributed among groups for both mtDNA (89.94%, *p*<0.05) and microsatellites (5.29%, *p*<0.05), but non-significant variation among populations within groups when the hypothesis of a northern versus southern Benguela subsystem population structure was tested.

**Figure 2:**
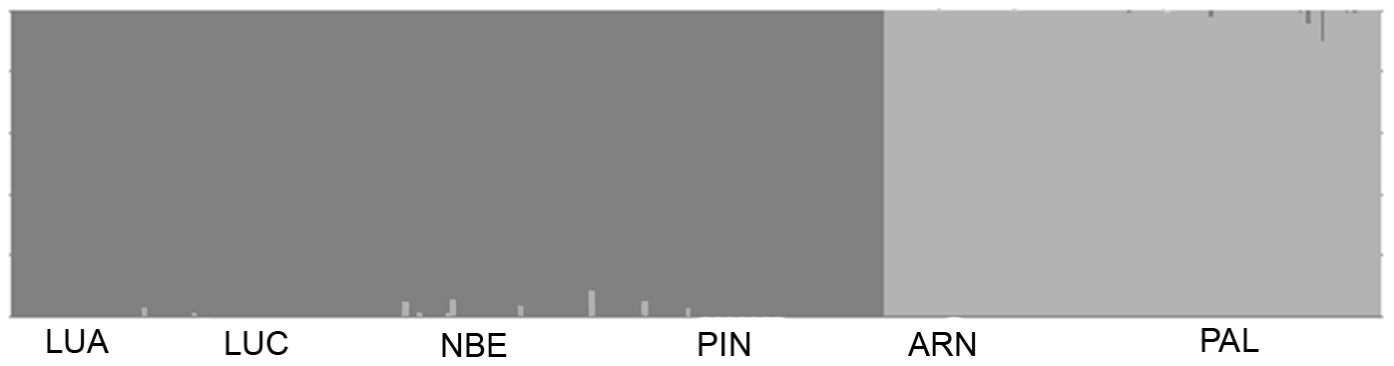
Number of genetic clusters observed within *A. aequidens* populations across the Benguela region. Assignment values for each individual fish obtained from STRUCTURE, based on genotypes from eight nuclear microsatellite loci, for *K* = 2. Cluster 1 (dark grey) are all northern (Angola) population fish; Cluster 2 (light grey) are all southern (South Africa) population fish.

Bayesian analyses of nuclear genetic population structure identified two genetic clusters, corresponding to the Angolan and South African samples, with no admixed individuals found between clusters (Figure 2).

### Phylogeography and demographic history of *A. aequidens* across the Benguela system

**Figure 3:**
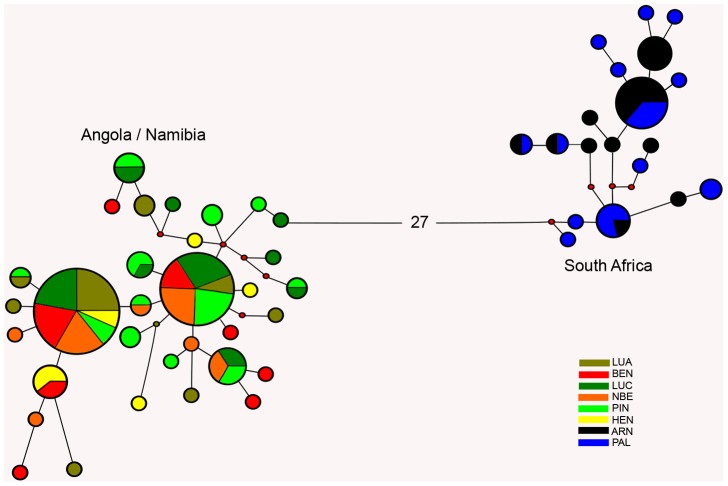
Haplotype network for *A. aequidens* sampled across the Benguela region. Haplotype network based on 583 bp of mtDNA CR sequences. Node sizes are proportional to number of individuals observed for that haplotype and colour codes refer to sampling site (see Table 1 for site designations). Red dots correspond to missing (non-sampled) haplotypes.

Reconstruction of haplotype relationships in *A. aequidens* revealed a clear phylogeographical pattern with two distinct haplogroups divergent by 27 mutational steps, consisting of individuals sampled in the northern Benguela region (LUA to HEN) and individuals sampled in the southern Benguela region (ARN and PAL) with no haplotypes shared between regions (Figure 3). The northern haplogroup comprised 32 haplotypes diverging by a maximum of 11 mutations, with haplotypes H1 and H2 occurring at equally high frequencies and observed from all sampling sites, most likely representing the ancestral type. Samples from BEN exhibited the largest number of private haplotypes, but there was no obvious geographical association among haplotypes (Figure 3). The structure of the southern haplogroup was very similar, with 19 haplotypes diverging by a maximum of nine mutations and with one central high frequency (i.e. ancestral) haplotype represented equally across the region, and no obvious geographical association among related haplotypes (Figure 3).

**Figure 4:**
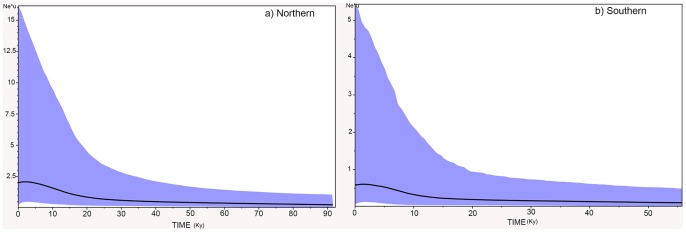
Bayesian Skyline Plots (BSPs) for the two populations of *A. aequidens* across the Benguela region. BSPs showing changes in effective population size (N_e_*μ) over time (KY). The solid line indicates the median estimate, and the 95% HPD interval is depicted in blue.

MtDNA data for both Angolan and South African populations revealed significant departures to neutrality expectation with Tajima's *D* and Fu's *FS* tests, and mismatch distribution analyses could not reject the null hypothesis of demographic expansion (Table 5). Estimates of time since expansion gave values of 27.41 Ka (95% CI: 5.24–99.90 Ka) and 24.53 Ka (95% CI: 14.63–135.32 Ka) for the northern and southern populations respectively (Table 5). Bayesian Skyline Plots also indicated demographic expansion in both populations (Figure 4), with estimates of time since expansion (10–50 Ka) similar to those obtained with the demographic model.

**Table 5:** Genetic demographic history for northern (Angola/northern Namibia) and southern (South Africa) Benguela subsystem populations of *A. aequidens*, based on mtDNA CR sequence variation: genetic diversity (*h* and *π*); neutrality tests (Tajima's *D* and Fu's *F_S_*); mismatch distribution parameters (*θ_0_* = population size before expansion in mutation units, *θ_1_* = population size after expansion in mutation units; and time since expansion (*T_exp_* in Ka; 95% CI in brackets).

	Northern	Southern
***h***	0.853	0.900
***π***	0.005	0.008
**D**	**−1.609**	−0.565
**FS**	**−20.775**	**−6.106**
**SSD**	0.006	0.008
**θ_0_**	1.239 (0.00–2.16)	1.374 (0.00–3.27)
**θ_1_**	7.317 (3.66 - ∞)	10.160 (5.61 - ∞)
**τ**	2.492 (0.63–8.02)	5.104 (1.08–13.42)
**T_exp_ (Ka)**	27.41 (5.24–99.90)	24.70 (14.63–135.32)

Statistically significant results (*p*<0.05) in bold.

Estimates of average, long-term and recent migration rates among the two putative populations of *A. aequidens* were very low, independent of the marker or mutation rate used. For mtDNA migration rates were estimated at *m_1_* = 0.0008 individuals from the southern (South Africa) to the northern (Angola) population, and *m_2_* = 0.0006 individuals in the opposite direction. Using microsatellite data, both MIGRATE and BAYESASS retrieved very similar results: *m_1_* = 0.0003 and *m_2_* = 0.0002 with a mutation rate of 1×10^−4^ per generation, and *m_1_* = 0.0019 and *m_2_* = 0.0074, respectively. These findings further support the results from STRUCTURE where no admixed individuals were identified between clusters (Figure 2). Coalescent-based estimates of effective population size (*N_e_*) and female effective population size (*N_ef_*) were high and of similar magnitude across the two populations (Angola *N_e_* = 2,550, *N_ef_* = 2,459; South Africa *N_e_* = 1,122, *N_ef_* = 2,462).

### Time since population divergence

Similar likelihoods were obtained for estimates of time since the most recent common ancestor (tmrca) between the two putative populations (Table 6), using the four methods. Estimates obtained with the standard mutation rate (3.6% per My) indicated that divergence occurred 1.57 Ma. Bayes Factor analyses indicated that the 2 Ma divergence scenario was twice as likely as the other hypotheses tested. The 2 Ma hypothesis gave a sequence divergence rate of 2.74% per My, closer to the estimated divergence rate for marine teleost CR, than those obtained with the other two biogeographical hypotheses (7% and 11% per My, respectively).

**Table 6:** Estimates of time since divergence between northern and southern subsystem *A. aequidens* populations: Ln(likelihood) – posterior likelihood of the calibration method employed; estimated time since most recent common ancestor (*tmrca* - Ma), and estimated divergence rate (% per My).

Calibration Method	Ln(likelihood)	Tmrca (Ma)	Estimated divergence rate
**3.6%**	−1456.221	1.574 (1.46–2.26)	-
**2 Ma**	−1457.064	2.380 (1.5–4.37)	2%
**1 Ma**	−1457.064	0.867 (0.5–1.53)	7%
**0.1 Ma**	−1457.064	0.467 (0.25–0.72)	11%

## Discussion

### Population connectivity across the Benguela Current region

Our results present evidence that *A. aequidens* around southern Africa is composed of two distinct and non-interbreeding populations, the distributions of which appear to coincide with contemporary oceanographic features of the Benguela Current system. There was no evidence of genetic population substructuring within the northern Benguela subsystem (Angola and northern Namibia) population or within the southern Benguela subsystem (South Africa) population, but high genetic divergence was found between the two populations in both mitochondrial and nuclear genomes.

The northern and southern Benguela boundary regions are characterized by multiple oceanographic features such as fronts (the Angola-Benguela frontal system in the north), freshwater outflows (the Cunene River on the Angola -Namibia border in the north, the Orange and Kei Rivers in the south), and multiple upwelling cells [17], [18]. Such features are known to limit dispersal of either early life history phases (eggs and larvae) and juveniles and/or adults of coastal fish and so disrupt contemporary gene flow [1], [5], [60]. These environmental features do not appear to significantly influence population connectivity in *A. aequidens* within each region, so effective long-term passive dispersal by eggs/larvae and/or active dispersal by adults must be occurring. The impact of oceanographic features in population substructuring is dependent on species life history features [60], so it is likely that the biological characteristics of *A. aequidens* may contribute to the observed panmixia. Both regional populations have pelagic eggs and an extended larval phase, likely to promote gene flow, but the two populations display divergent adult reproductive migration behavior that appears to be matched to different oceanographic regimes in the two subsystems (Henriques et al. submitted). Based on adult catch information and the small size at maturity, there is no evidence of a spawning migration in the northern population (Henriques et al. submitted). With a continuous distribution throughout the northern Benguela boundary region (W. Potts, unpubl), it appears that this population has evolved an extended spawning season (seven months) to ensure that eggs are widely distributed by the bi-directional currents in the region [61]. In contrast, the South African population appears to have evolved a short (two months) spawning season and annual reproductive migration to a single common spawning ground off the coast of KwaZulu-Natal [30]. Broad egg and larval dispersal is facilitated from this spawning site by the southwest flowing Agullhas Current system [62]. The divergent reproductive strategies maintain single widespread genetic populations in both regions, possibly an adaptation to increase dispersal and maximize survival in these unstable and fluctuating boundary environments.

Given the effectively homogenized population structure within each region, the substantial genetic divergence between northern and southern populations of *A. aequidens* must be linked to a major impermeable barrier to gene flow located between the two putative populations. In the case of southern African *A. aequidens*, the complete isolation of the two populations, indicated by high levels of population divergence for both mtDNA and nuclear markers, combined with an absence of shared haplotypes or identifiable migrants (migration rates less than 1% per generation), suggest not only that the Benguela Current prevents dispersion in all life stages, but also that this is a long term and on-going effect. The genetic break between northern and southern populations coincides with the main upwelling features associated with the Benguela system, with several upwelling cells along southern Namibian and western South African coasts [17], and particularly the perennial Lüderitz upwelling cell off central Namibia [16], [17], [18]. Low sea surface temperatures associated with these upwelling cells and the narrow continental shelf in this region are hypothesized to have prevented gene flow between the northern and southern populations. Upwelling cells are known to induce local larval retention to either side, by disrupting longshore transport and driving pelagic eggs and larvae offshore where the environment is unsuitable for survival and/or recruitment [7], [63]. Cold water temperatures would discourage survival, migration/dispersal and effective reproduction by the juveniles and adults of this species, due to its warm-temperature requirements [30]. The majority of upwelling cells in the Benguela region are non-perennial, which could allow dispersal by adults between upwelling events. Therefore, it is most likely that the permanent Lüderitz upwelling cell constitutes the impermeable barrier to the transport of the early-life history stages and spread of juveniles and adults of *A. aequidens* across the Benguela system.

Population genetic divergence values for the pairwise comparisons between samples from the northern and southern populations (mtDNA *φ*_ST_ = 0.902, microsatellite *F*_ST_ = 0.055: probability of genetic homogeneity for both = *p*<0.001) were very high for a marine teleost. Marine fish typically have widespread distributions, historically large effective population sizes, high dispersal potential and high fecundity, all of which features promote low average genetic differentiation [64], even if species occur across oceanographic systems that can potentially constitute barriers to gene flow [3], [6], [60]. The genetic divergence observed here is more similar to that reported for deep vicariant events [65], such as those associated with the uplifting of the Isthmus of Panama [66], and suggest that isolation of *A. aequidens* in the Benguela Current region is linked to the presence of an ancient and impermeable barrier to gene flow.

### Past population history inferred from present diversity

The genetic analyses of demographic changes detected significant fluctuations in population growth, migration rate and effective population size, in both regional populations of *A. aequidens*, likely reflecting historical environmental changes in the Benguela Current region. Coalescent-based estimates of long-term effective population size and female effective population size were very similar for the two populations, and very similar in range to those reported for other widespread marine fish [67].

The mtDNA data (assuming selective neutrality) indicated strong support for past population growth, suggesting that both *A. aequidens* populations have undergone significant expansion pre-dating, or during, the last glacial maximum (LGM) around 25 Ka. As estimates of time since expansion based solely on mismatch distribution parameters have severe drawbacks as the only source of information regarding a species' demographic history, as they may be influence by early branching in the gene tree [65], the use of alternative estimators such as coalescent-based Bayesian Skyline Plots (BSP) can provide corroboration of expansion times. Although estimates of timing of expansion should not be interpreted at face-value due to commonly large 95% confidence intervals, the obtained results in this study based both on mismatch distributions and BSP, combined with extant population genetic diversity, suggested that population expansion occurred earlier and/or from a larger starting size in the northern than in the southern *A. aequidens* population. Severe declines in many marine fishes are associated with the LGM, with the majority of populations showing recovery after the end of the last glacial period ∼10 Ka [13], [68], [69]. Population expansion in *A. aequidens* does not appear to have been as rapid, but occurred earlier than reported for other fishes. In particular, studies conducted on other species in the southern Benguela region suggest that population growth occurred quite recently, during the Holocene (8-6 Ka) [25], [26], [27], [28]. Even accounting for the large confidence intervals commonly associated with such estimates, an earlier time since expansion is implied for both *A. aequidens* populations than those reported for other species in the Benguela Current region. The timing and rate of *A. aequidens* population expansion suggests that both populations may have survived the LGM in warm refugia that allowed range and population expansion from early in the interglacial period. Early population expansions pre-dating the end of the LGM have been described in warm-temperate fish species in the northern hemisphere, commonly associated with suitable refugia [67], [70]. Despite reduced sea surface temperatures (2°C to 3°C cooler) [23] and sea level (<100 m than present day) [71] associated with the LGM, which combined with a narrow continental shelf may have severely reduced the number and size of suitable habitats, the presence of the tropical Angola and Agulhas currents to either side of the Benguela Current are likely to have contributed to the maintenance of suitable habitats [27] for a warm-temperate benthopelagic species such as *A. aequidens*. Furthermore, a warming period between 40-18 Ky has been reported for the region, which may have contributed to an earlier population expansion of warm-temperate species in the Benguela Current system [72].

### Timing of isolation of northern and southern *A. aequidens* populations

The concordance between mitochondrial and nuclear genetic markers showing substantial differentiation, and the lack of indication of any contemporary gene flow, indicates that isolation and divergence of the northern and southern Benguela populations of *A. aequidens* occurred deep in the past and has continued to the present day.

The coalescent simulations indicated that the isolation of *A. aequidens* populations in the Benguela Current region dates from the Pliocene-Pleistocene transition approximately 2 Ma. This transition was characterized by a generalized cooling of world oceans and major changes to circulation patterns, which have been linked to population isolation and vicariant speciation events for many fishes [14]. In the Benguela Current region, intensification of the upwelling regime and establishment of the present day characteristics of the system (including the perennial Lüderitz upwelling) occurred during the Pliocene-Pleistocene transition, suggesting that the upwelling cell was responsible for the initial, as well as continuing, isolation of *A. aequidens* populations.
